# Case Report: Neutrophil extracellular trap formation in palisaded neutrophilic granulomatous dermatitis

**DOI:** 10.3389/fimmu.2026.1906446

**Published:** 2026-09-01

**Authors:** Lisa Minai, Youichi Ogawa, Yoshiaki Kobayashi, Takuya Sato, Daiki Nakagomi, Shinji Shimada, Tatsuyoshi Kawamura

**Affiliations:** 1 Department of Dermatology, Faculty of Medicine, University of Yamanashi, Yamanashi, Japan; 2 Department of Rheumatology, Faculty of Medicine, University of Yamanashi, Yamanashi, Japan

**Keywords:** M1 M2 macrophage phenotype, macrophages, neutrophil extracellular traps (NET), neutrophils, palisaded neutrophilic granulomatous dermatitis, reactive granulomatous dermatitis, rheumatoid arthritis (RA)

## Abstract

Palisaded neutrophilic granulomatous dermatitis (PNGD) is an uncommon reactive granulomatous dermatosis frequently associated with rheumatoid arthritis (RA). Histopathologically, PNGD is characterized by neutrophilic infiltrates, degenerated collagen bundles, and surrounding palisading histiocytes; however, the contribution of neutrophils to lesion formation remains poorly understood. We report two patients with RA-associated PNGD who presented with chronic erythematous papules and nodules on the elbows. Histopathological examination revealed characteristic palisaded granulomatous inflammation with dense neutrophilic aggregates and degenerated collagen. Immunofluorescence studies demonstrated abundant neutrophil extracellular trap (NET) formation within the neutrophilic aggregates. NETs are extracellular chromatin structures released by activated neutrophils that can promote sterile inflammation and tissue injury. In both cases, CD68-positive macrophages surrounded the NET-rich structures. As reported, M2 macrophages were observed throughout the lesions; however, macrophages directly contacting NET-rich aggregates showed little or no expression of the M2- and M1-associated markers, suggesting that they did not exhibit a conventional M1 or M2 macrophage profile. Further characterization revealed that these peri-NET cells exhibited a CD68-dominant/CD14-low/HLA-DR-low phenotype. To our knowledge, these findings provide the first documentation of NET formation within PNGD lesions. Comparative studies are required to determine whether this finding is enriched in or specific to PNGD. We hypothesize that repetitive mechanical stress, together with RA-associated alterations in NET formation and clearance, may favor the persistence of NET-rich inflammatory foci. Overall, the documentation of NET formation and its spatial association with macrophages expands the histopathological characterization of PNGD.

## Introduction

Palisaded neutrophilic granulomatous dermatitis (PNGD) was originally reported in 1965 in patients with rheumatoid arthritis (RA) as an unusual form of rheumatoid granuloma, with histologic findings resembling the palisaded granulomas seen in rheumatoid nodules (1). PNGD is now recognized as an uncommon form of reactive granulomatous dermatitis that is frequently associated with autoimmune diseases, particularly RA. Clinically, PNGD typically presents as symmetrical erythematous to skin-colored papules or nodules on extensor surfaces, especially the elbows and dorsal fingers. Histopathologically, PNGD is characterized by dense neutrophilic infiltrates, leukocytoclastic debris, degenerated collagen bundles, and surrounding palisading histiocytes (2, 3). Increasing evidence suggests that PNGD represents a reactive tissue response rather than a distinct disease entity and may belong to a broader spectrum of reactive granulomatous dermatitis (4–6).

Although neutrophils constitute a central component of PNGD lesions, the mechanisms by which they contribute to lesion formation remain poorly understood. Neutrophil extracellular traps (NETs) are extracellular chromatin structures decorated with neutrophil-derived proteins such as neutrophil elastase and myeloperoxidase. NETs play important roles in host defense but can also induce tissue injury and perpetuate sterile inflammation (7, 8). In RA, circulating and tissue-infiltrating neutrophils exhibit an increased propensity for NET formation, and NETs are increasingly recognized as important contributors to disease pathogenesis (9). Beyond RA, NET formation has also been documented in a growing number of neutrophilic dermatoses, including pyoderma gangrenosum, Sweet syndrome, hidradenitis suppurativa, and pustular dermatoses (10, 11). However, the biological effects of NETs may depend on their spatial organization and the local neutrophil density. At high neutrophil densities, individual NETs can combine to form aggregated NETs (aggNETs), which can acquire inflammation-limiting properties by degrading cytokines and chemokines and by sequestering and detoxifying potentially cytotoxic extracellular histones (12, 13). PNGD lesions characteristically contain dense neutrophilic aggregates surrounded by histiocytes; however, whether NETs are present within these neutrophilic aggregates and what role they may play in PNGD remain unknown.

Here, we describe two patients with RA-associated PNGD and document abundant NET formation within the central neutrophilic aggregates of their lesions. We also characterize the phenotype of macrophages surrounding these NET-rich structures and discuss the potential relationships among NET formation and macrophage phenotype in PNGD.

## Case description

**Figure 1:**
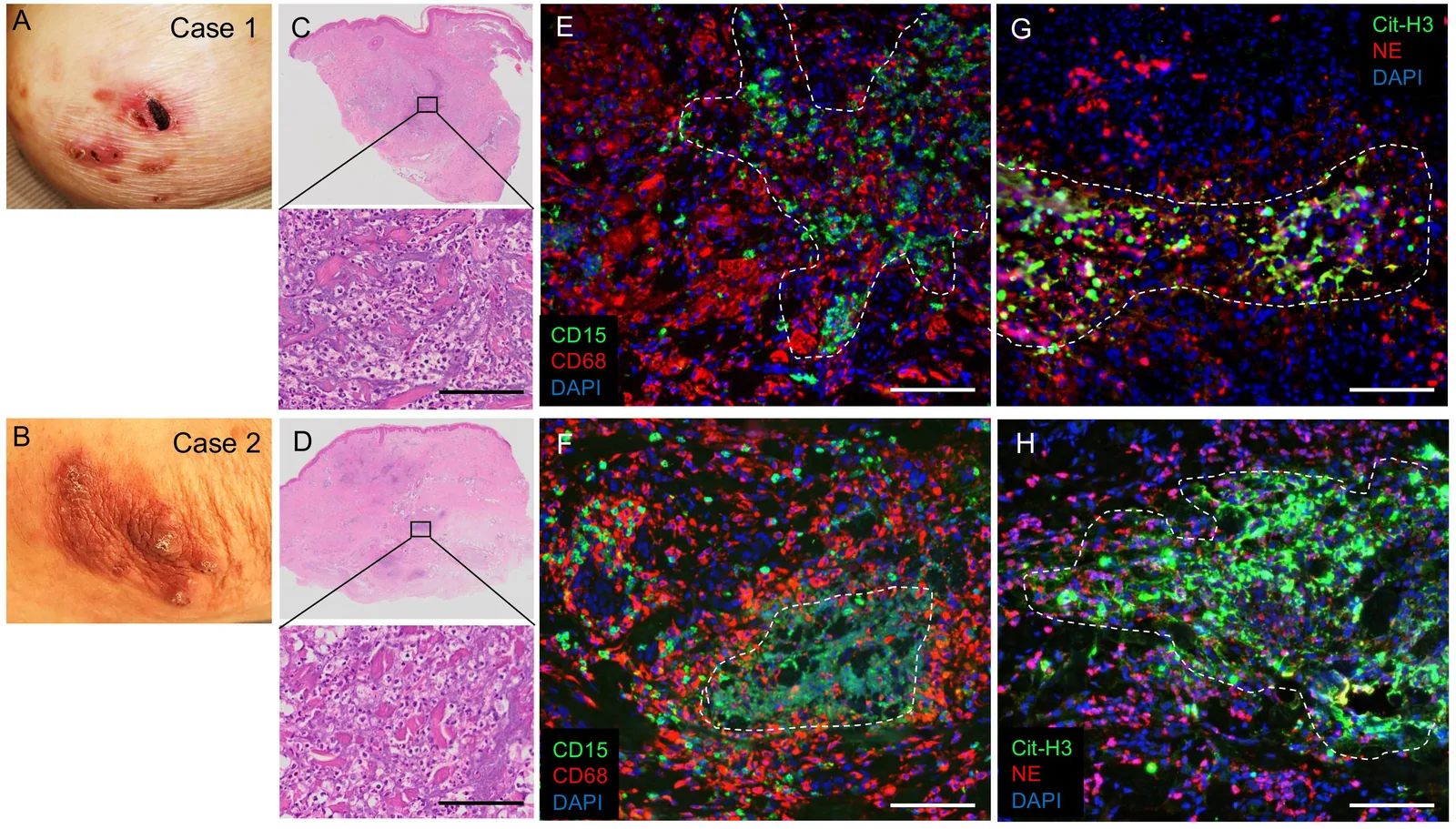
Clinical, histopathological, and immunofluorescence findings of PNGD lesions. Clinical presentation of case 1 **(A)** and case 2 **(B)**. Histopathological examination of case 1 **(C)** and case 2 **(D)** (hematoxylin–eosin staining). Immunofluorescence staining for CD15 (green) and CD68 (red) of case 1 **(E)** and case 2 **(F)**. Immunofluorescence staining for citrullinated histone H3 (cit-H3; green) and neutrophil elastase (NE; red) of case 1 **(G)** and case 2 **(H)**. Original magnifications: **(C, D)** ×10 and ×100, **(E–H)** ×400. Scale bars, 100 μm. The following primary antibodies were used for immunofluorescence staining: a rabbit monoclonal anti-CD15 antibody (clone SP159; Abcam, Cambridge, UK), a mouse monoclonal anti-CD68 antibody (clone 514H12; Bio-Rad Laboratories, Hercules, CA, USA), a rabbit polyclonal anti-histone H3 antibody (citrulline R2 + R8 + R17; Abcam, Cambridge, UK), and a mouse monoclonal anti- NE antibody (clone 950317; R&D Systems, Minneapolis, MN, USA). Samples were mounted and counterstained with VECTASHIELD Vibrance Antifade Mounting Medium with DAPI (catalog no. H-1800-10; Vector Laboratories, Newark, CA, USA).

Case 1: A 64-year-old woman with a history of RA, diabetes mellitus, and liver failure presented with painful erythematous nodules on both elbows. Because of severe RA-associated joint deformities and pain, she relied on a wheelchair and frequently used her elbows to support her body weight during transfers. The lesions had been present for several years and were predominantly located at sites repeatedly exposed to pressure from the wheelchair armrests (**Figure 1A**). Case 2: A 62-year-old man with RA presented with symmetrical erythematous papules on both elbows that had persisted for several years (**Figure 1B**).

In both patients, skin biopsy specimens were obtained from representative elbow lesions. Histopathological examination revealed dense aggregates of neutrophils associated with nuclear debris and degenerated collagen bundles in the middle-to-deep dermis. These structures were surrounded by prominent palisading infiltrates of histiocytes, consistent with the diagnosis of PNGD (**Figures 1C, D**). Direct immunofluorescence analysis of Case 1 showed no specific deposition of IgG or C3 within the PNGD lesion (**Supplementary Figure 1**).

To further characterize the cellular composition of the lesions, immunofluorescence studies were performed. CD15-positive neutrophils formed compact aggregates that were surrounded by CD68-positive macrophages (**Figures 1E, F**). Interestingly, extracellular web-like structures within the neutrophilic aggregates showed co-localization of citrullinated histone H3 and neutrophil elastase (**Figures 1G, H**), together with DAPI-positive extracellular chromatin in the high-magnification images (**Supplementary Figure 2**), supporting their identification as NETs.

**Figure 2:**
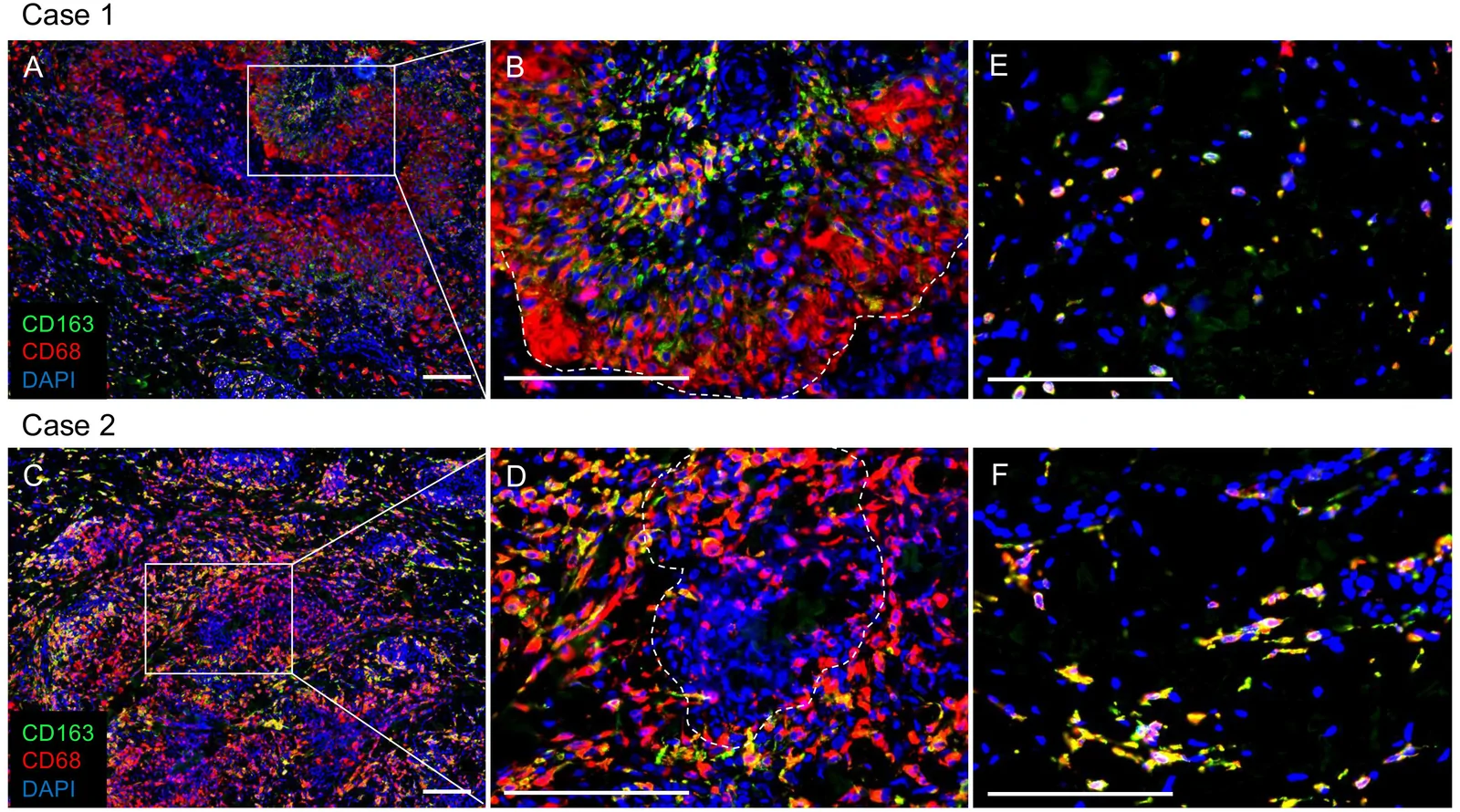
Characterization of infiltrating M2 macrophages. Immunofluorescence staining for CD163 (green) and CD68 (red) of case 1 **(A, B)** and case 2 **(C, D)** in the lesional skin. Immunofluorescence staining for CD163 (green) and CD68 (red) of case 1 **(E)** and case 2 **(F)** in the perilesional skin. Original magnifications: **(A, C)** ×100, **(B, D–F)** ×400. Scale bars, 100 μm. The following primary antibodies were used for immunofluorescence staining: a rabbit monoclonal anti-CD163 antibody (clone EPR19518; Abcam, Cambridge, UK) and a mouse monoclonal anti-CD68 antibody (clone 514H12; Bio-Rad Laboratories, Hercules, CA, USA). Samples were mounted and counterstained with VECTASHIELD Vibrance Antifade Mounting Medium with DAPI (catalog no. H-1800-10; Vector Laboratories, Newark, CA, USA).

**Figure 3:**
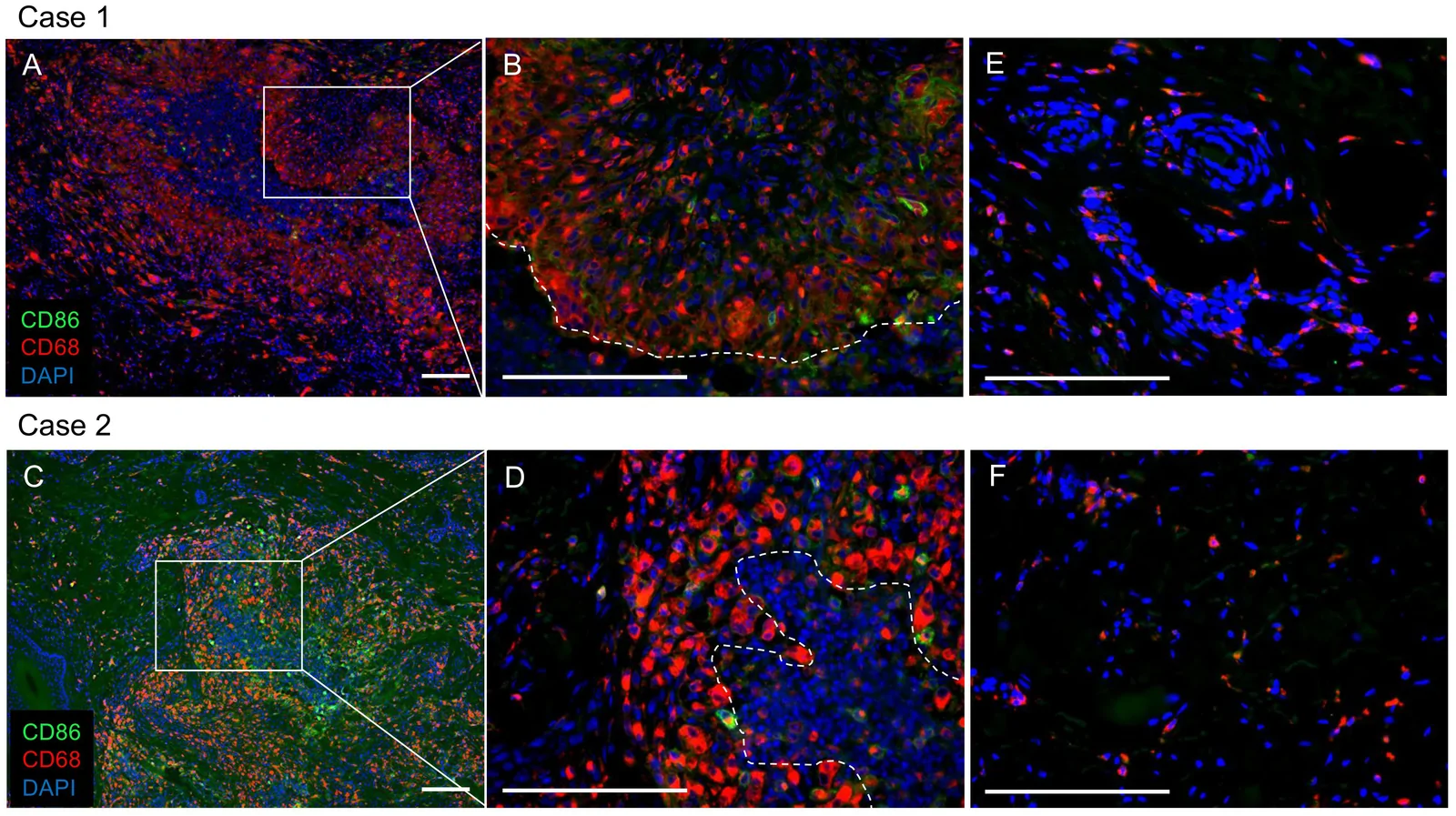
Characterization of infiltrating M1 macrophages. Immunofluorescence staining for CD86 (green) and CD68 (red) of case 1 **(A, B)** and case 2 **(C, D)** in the lesional skin. Immunofluorescence staining for CD86 (green) and CD68 (red) of case 1 **(E)** and case 2 **(F)** in the perilesional skin. Original magnifications: **(A, C)** ×100, **(B, D–F)** ×400. Scale bars, 100 μm. The following primary antibodies were used for immunofluorescence staining: a rabbit monoclonal anti-CD86 antibody (clone E2G8P; Cell Signaling Technology, Danvers, MA, USA) and a mouse monoclonal anti-CD68 antibody (clone 514H12; Bio-Rad Laboratories, Hercules, CA, USA). Samples were mounted and counterstained with VECTASHIELD Vibrance Antifade Mounting Medium with DAPI (catalog no. H-1800-10; Vector Laboratories, Newark, CA, USA).

Because macrophages constitute a major cellular component of PNGD lesions, we next analyzed their phenotype. Consistent with a previous report (14), numerous CD163-positive macrophages were observed throughout the lesions; we additionally found widespread expression of CD206 (**Figures 2A–D**; **Supplementary Figures 3A–D**). However, detailed examination demonstrated that macrophages directly contacting NET-rich neutrophil aggregates were largely negative for CD163 and CD206 (**Figures 2A–D**; **Supplementary Figures 3A–D**). These macrophages also showed little or no expression of the M1-associated marker CD86 and CD80 (**Figures 3A–D**; **Supplementary Figures 4A–E**), indicating that they could not be classified as conventional M1 or M2 macrophages. To quantitatively assess this spatial heterogeneity, we defined CD68-positive cells directly contacting NET-positive neutrophilic aggregates as peri-NET CD68-positive cells and those located farther from the NET-rich aggregates, but within the surrounding histiocytic infiltrates, as NET-distal CD68-positive cells (**Supplementary Figure 5A**). Quantification of five ×400 fields from each region in each case demonstrated that the proportions of CD163- and CD206-positive cells were significantly lower among peri-NET CD68-positive cells than among NET-distal CD68-positive cells in both cases (p < 0.001; **Supplementary Figure 5B**). These findings demonstrate a spatially distinct macrophage marker profile associated with proximity to NET-rich neutrophilic aggregates.

By contrast, in the perilesional skin, outside both the NET-rich neutrophilic aggregates and the surrounding histiocytic infiltrates, macrophages predominantly expressed CD163 and CD206 (**Figures 2E, F**; **Supplementary Figures 3E, F**), but not CD86 and CD80 (**Figures 3E, F**; **Supplementary Figures 4F, G**), consistent with an M2-associated marker profile. In addition, macrophages infiltrating the neutrophil aggregates appeared larger and more hypertrophic than those in adjacent areas and exhibited prominent cytoplasmic extensions suggestive of cellular activation and interaction with NET-containing structures (**Figures 2**, **3**).

We next characterized the CD68-positive peri-NET cells, which showed little or no expression of the M1- and M2-associated markers examined. High-magnification examination of hematoxylin and eosin–stained sections demonstrated that these cells exhibited histiocyte-like morphology and formed an interface surrounding the NET-rich neutrophilic aggregates (**Supplementary Figure 6**).

To further characterize the lineage-associated phenotype of the peri-NET cells, we examined CD14 and CD68 expression. Because CD14 is predominantly localized to the plasma membrane, whereas CD68 is primarily localized to intracellular lysosomal and endosomal membranes, their expression was evaluated at the cellular level rather than on the basis of direct fluorescence colocalization. The histiocyte-like cells forming the immediate interface with the NET-rich aggregates showed strong CD68 staining but weak or undetectable CD14 staining (**Supplementary Figures 7A–D**). By contrast, CD14-dominant cells with weaker CD68 staining were scattered within and around the NET-rich aggregates but did not form the palisading cellular interface. Similar CD68-dominant and CD14-dominant cell populations were present farther from the NET-rich aggregates and in the perilesional skin (**Supplementary Figures 7E, F**). Thus, although CD14-dominant monocyte-like cells were distributed throughout the lesions, the cells immediately surrounding the NET-rich aggregates predominantly exhibited a CD68-dominant/CD14-low phenotype, further supporting their histiocytic/macrophage-like identity.

We subsequently evaluated HLA-DR expression as a marker associated with antigen presentation. The CD68-positive peri-NET cells showed weak or virtually undetectable HLA-DR expression (**Supplementary Figures 8A–D**), whereas CD68-positive macrophages in the perilesional skin expressed HLA-DR (**Supplementary Figures 8E, F**). Collectively, despite their histiocyte-like morphology and CD68 expression, the peri-NET cells showed little or no expression of CD14, CD80, CD86, CD163, CD206, or HLA-DR. These findings identify a spatially restricted CD68-positive histiocytic population that lacked the conventional monocyte-associated, M1-associated, M2-associated, and antigen-presentation-associated marker profiles examined.

## Discussion

PNGD is a rare reactive granulomatous dermatosis that is most commonly associated with RA and other autoimmune diseases. Although the histopathological features of PNGD have been well characterized, the mechanisms responsible for the accumulation of neutrophils and the formation of palisaded granulomatous lesions remain poorly understood. In the present cases, abundant NET formation was observed within the characteristic neutrophilic aggregates of PNGD lesions. NETs have previously been demonstrated in a broad range of neutrophilic dermatoses (10, 11). However, to our knowledge, NET formation in PNGD has not previously been reported. Thus, the novelty of the present observations lies specifically in documenting NET formation within the characteristic neutrophilic aggregates of PNGD.

The predilection of PNGD for pressure-bearing sites may provide a clue to its pathogenesis. Both patients developed lesions on the extensor surfaces of the elbows, a location repeatedly exposed to mechanical stress. Mechanical injury induces the release of damage-associated molecular patterns, including extracellular ATP, which acts as a potent chemoattractant for neutrophils through purinergic receptors. In addition, extracellular ATP has been shown to promote NET formation (15, 16). Therefore, repetitive mechanical stimulation may establish a microenvironment that favors the recruitment and activation of neutrophils at specific anatomical sites. Notably, Case 1 was subsequently hospitalized for four weeks because of a severe soft-tissue infection of the right lower extremity and received antibiotic therapy. Throughout hospitalization, she remained on bed rest, thereby avoiding the habitual mechanical pressure on her elbows associated with wheelchair transfers. No treatment was administered specifically for the PNGD lesions. Nevertheless, the elbow lesions markedly regressed by the fourth week of hospitalization (**Supplementary Figure 9**). This clinical course provides additional, albeit indirect, support for the hypothesis that repetitive mechanical stress contributes to the localization and persistence of PNGD lesions.

The strong association between PNGD and RA may further amplify this process. A systematic review demonstrated that nearly half of patients with PNGD have underlying autoimmune diseases, with RA being the most frequent comorbidity (3). Neutrophils from patients with RA are known to exhibit an enhanced propensity for spontaneous NETosis (9). In addition to enhanced NET formation, reduced serum DNase I activity has been reported in patients with RA, raising the possibility that impaired extracellular DNA degradation contributes to NET persistence (17). Collectively, we hypothesize that mechanical stress-induced neutrophil recruitment, together with RA-associated enhancement of NET formation and impairment of NET clearance, may favor the persistence of NET-rich inflammatory foci.

A notable finding was the spatially restricted phenotype of macrophages at the interface with the NET-rich aggregates. Compared with macrophages located farther from these aggregates, peri-NET cells exhibited histiocyte-like morphology with a CD68-dominant/CD14-low/HLA-DR-low profile and little or no expression of the M1- or M2-associated markers examined. Previous studies have shown that NETs can activate macrophages and modify their cytokine profiles during sterile inflammation (18). Extracellular ATP released following tissue injury can also influence macrophage activation through P2X7 receptor signaling (19). Collectively, these observations raise the possibility that NETs and extracellular ATP influence the phenotype of macrophages within the local inflammatory microenvironment of PNGD.

The unusual marker profile of the peri-NET cells may also be related to their close spatial association with the dense NET-rich aggregates. At high neutrophil densities, individual NETs can combine to form aggregated NETs (aggNETs), which contain proteolytically active neutrophil serine proteases. Previous studies have demonstrated that aggNETs degrade inflammatory cytokines and chemokines (12). They also sequester and detoxify potentially cytotoxic extracellular histones (13). This proteolytic activity limits neutrophil recruitment and activation, thereby promoting the resolution of neutrophilic inflammation. Although the NET-rich aggregates observed in PNGD were not established as bona fide aggNETs, they may create a similarly protease-rich and inflammation-modulating microenvironment. We hypothesize that local degradation or sequestration of soluble mediators within this microenvironment could attenuate the signals that maintain conventional monocyte-associated, macrophage-polarization, and antigen-presentation-associated marker profiles, potentially contributing to the CD68-dominant/CD14-low/HLA-DR-low phenotype of the peri-NET cells. The presence of CD14-dominant cells and HLA-DR-positive macrophages farther from the NET-rich aggregates is consistent with, but does not establish, a spatially restricted local effect. Because the present study did not assess protease activity, cytokine degradation, or the direct effects of NET-rich aggregates on macrophages, whether these structures are functionally equivalent to aggNETs and whether they directly induce the observed macrophage phenotype remain to be determined.

Several limitations should be acknowledged. First, we did not examine comparator lesions, such as rheumatoid nodules, interstitial/reactive granulomatous dermatitis, or other neutrophil-rich dermatoses. Because NET formation has been described in various inflammatory and neutrophilic skin diseases, the presence of NETs in our cases may reflect the dense accumulation of neutrophils within the lesions and/or the systemic predisposition to NET formation associated with RA, rather than a process specific to PNGD. Second, Case 1 had diabetes mellitus and liver failure, both of which may influence neutrophil and NET biology. Neutrophils from patients with diabetes have been reported to be primed for NET formation (20), whereas increased circulating NET markers have been observed in patients with advanced liver dysfunction (21). These comorbidities may therefore have contributed to the prominent NET formation observed in Case 1. Accordingly, our findings establish the presence of NETs in the examined PNGD lesions but do not demonstrate their specificity for PNGD or establish a causal role in its pathogenesis.

## Conclusion

In two patients with RA-associated PNGD, we documented abundant NET formation within the characteristic neutrophilic aggregates and identified a spatially distinct population of peri-NET histiocytes exhibiting a CD68-dominant/CD14-low/HLA-DR-low phenotype with little or no expression of the M1- and M2-associated markers examined. These observations expand the histopathological characterization of PNGD by revealing not only NET formation but also a distinctive organization of macrophages at the interface with NET-rich aggregates. Together, our findings raise the possibility that NET-rich neutrophilic foci and locally altered macrophage phenotypes represent linked components of the palisaded inflammatory architecture of PNGD. Comparative and functional studies will be required to determine whether these findings are enriched in PNGD and whether NET-rich aggregates directly influence the phenotype of surrounding macrophages.

## Data Availability

The original contributions presented in the study are included in the article/**Supplementary Material**. Further inquiries can be directed to the corresponding author.
