# Supplementary material for: Case Report: Neutrophil extracellular trap formation in palisaded neutrophilic granulomatous dermatitis
Source: Front Immunol. 2026 Sep 1;17:1906446. doi: 10.3389/fimmu.2026.1906446 (PMC13575740; doi:10.3389/fimmu.2026.1906446)
Supplement: Supplementary file 1 [file SupplementaryFile1.pdf]

Supplementary Figure 1

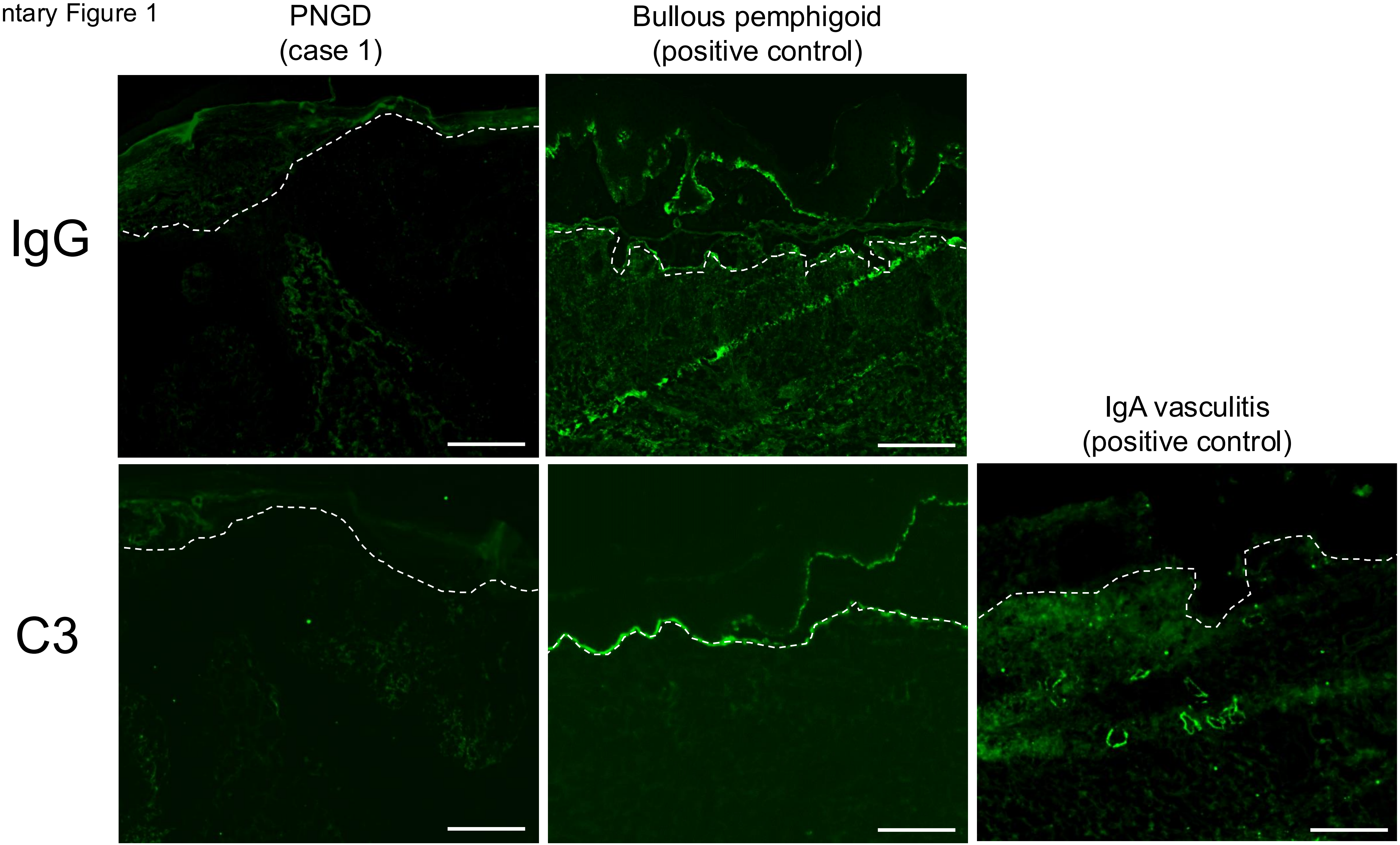

Supplementary Figure 1. Direct immunofluorescence findings in a PNGD lesion. Direct immunofluorescence staining of frozen tissue sections was performed using FITC-conjugated polyclonal rabbit anti-human IgG and FITC-conjugated polyclonal rabbit anti-human complement C3c antibodies (both from Agilent Technologies, Santa Clara, CA, USA). Analysis of lesional skin from Case 1 showed no specific deposition of IgG or C3. A bullous pemphigoid specimen used as a positive control showed linear deposition of IgG and C3 along the dermal–epidermal junction. An IgA vasculitis specimen used as an additional positive control for C3 showed deposition within the walls of dermal vessels. The dashed lines indicate the dermal–epidermal junction or dermal vessel walls. Scale bars, 200 μm.

Supplementary Figure 2

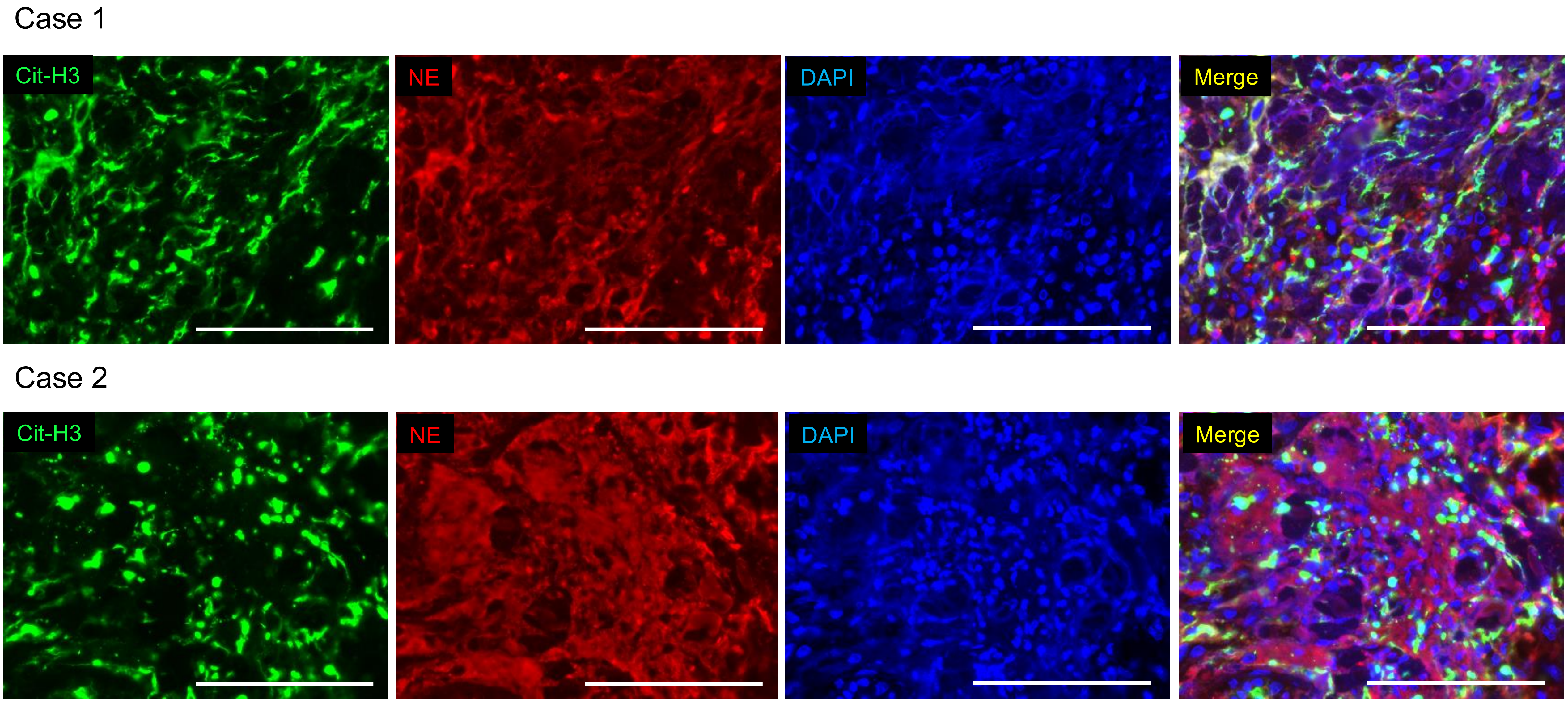

Supplementary Figure 2. High-magnification immunofluorescence images demonstrating extracellular DNA in NET-like structures within PNGD lesions. Representative high-magnification images from Case 1 (upper row; corresponding to Figure 1G) and Case 2 (lower row; corresponding to Figure 1H) are shown. Citrullinated histone H3 (cit-H3; green), neutrophil elastase (NE; red), DAPI (blue), and merged images are presented separately. The extracellular web-like cit-H3- and NE-positive structures co-localized with DAPI-positive extracellular chromatin, supporting their identification as NETs. Scale bars, 100 µm. The following primary antibodies were used for immunofluorescence staining: a rabbit polyclonal anti-histone H3 antibody (citrulline R2 + R8 + R17; Abcam, Cambridge, UK) and a mouse monoclonal anti- NE antibody (clone 950317; R&D Systems, Minneapolis, MN, USA). Samples were mounted and counterstained with VECTASHIELD Vibrance Antifade Mounting Medium with DAPI (catalog no. H-1800-10; Vector Laboratories, Newark, CA, USA).

Supplementary Figure 3

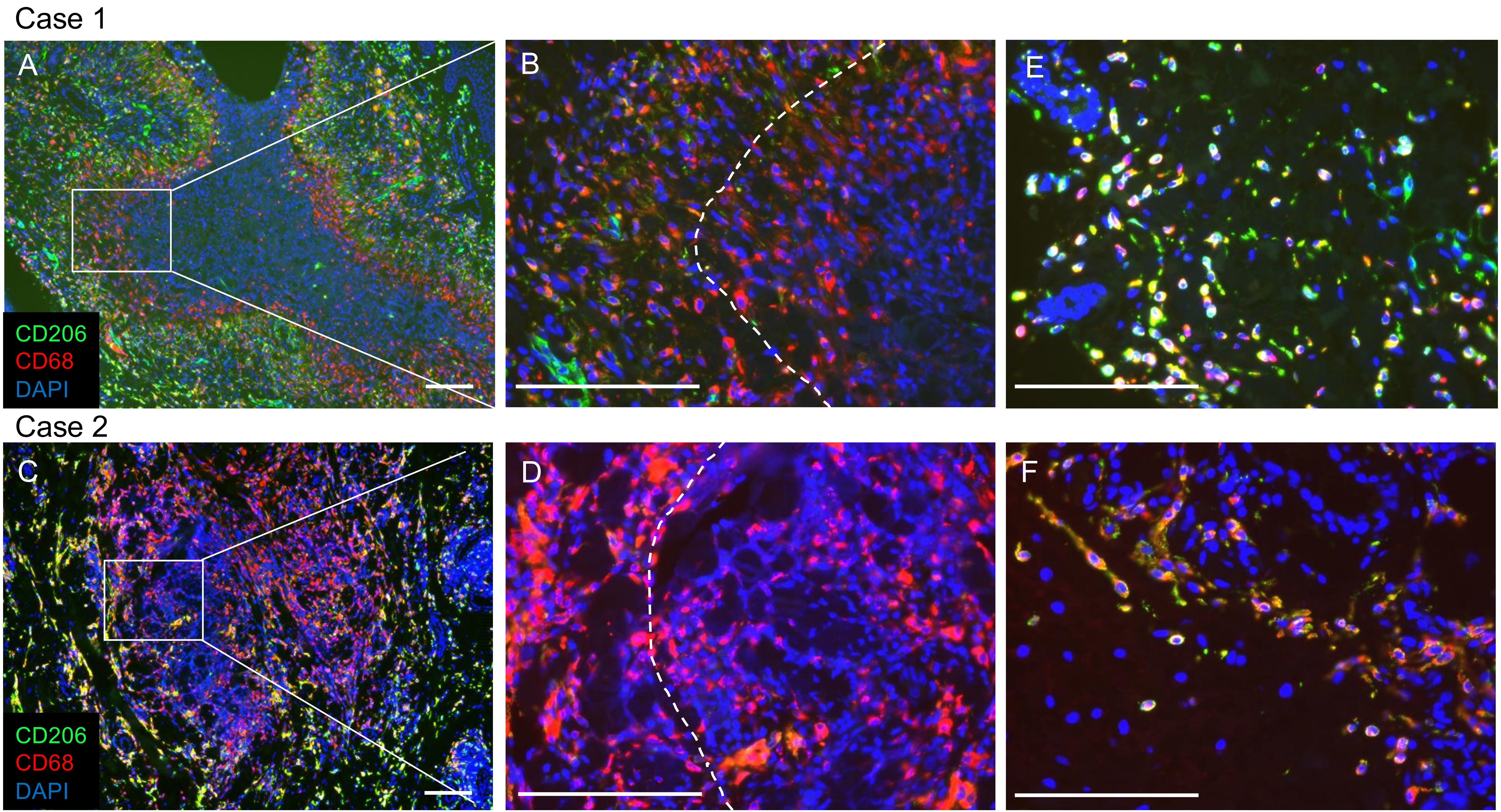

Supplementary Figure 3. CD206 expression in peri-NET cells. Representative immunofluorescence staining for CD206 (green) and CD68 (red), with DAPI nuclear counterstaining (blue), in lesional and perilesional skin from patients with PNGD. Low-magnification images of the lesional skin from case 1 (A) and case 2 (C) are shown, with the boxed regions presented at higher magnification in B and D, respectively. Dashed lines delineate the NET-rich neutrophilic aggregates. Perilesional skin from case 1 (E) and case 2 (F) is also shown. Original magnifications: (A and C) ×100; (B, D, E, and F) ×400. Scale bars, 100 μm. The following primary antibodies were used: a rabbit polyclonal anti-CD206 antibody (catalog no. ab64693; Abcam, Cambridge, UK) and a mouse monoclonal anti-CD68 antibody (clone 514H12; Bio-Rad Laboratories, Hercules, CA, USA). Samples were mounted and counterstained using VECTASHIELD Vibrance Antifade Mounting Medium with DAPI (catalog no. H-1800-10; Vector Laboratories, Newark, CA, USA).

Supplementary Figure 4

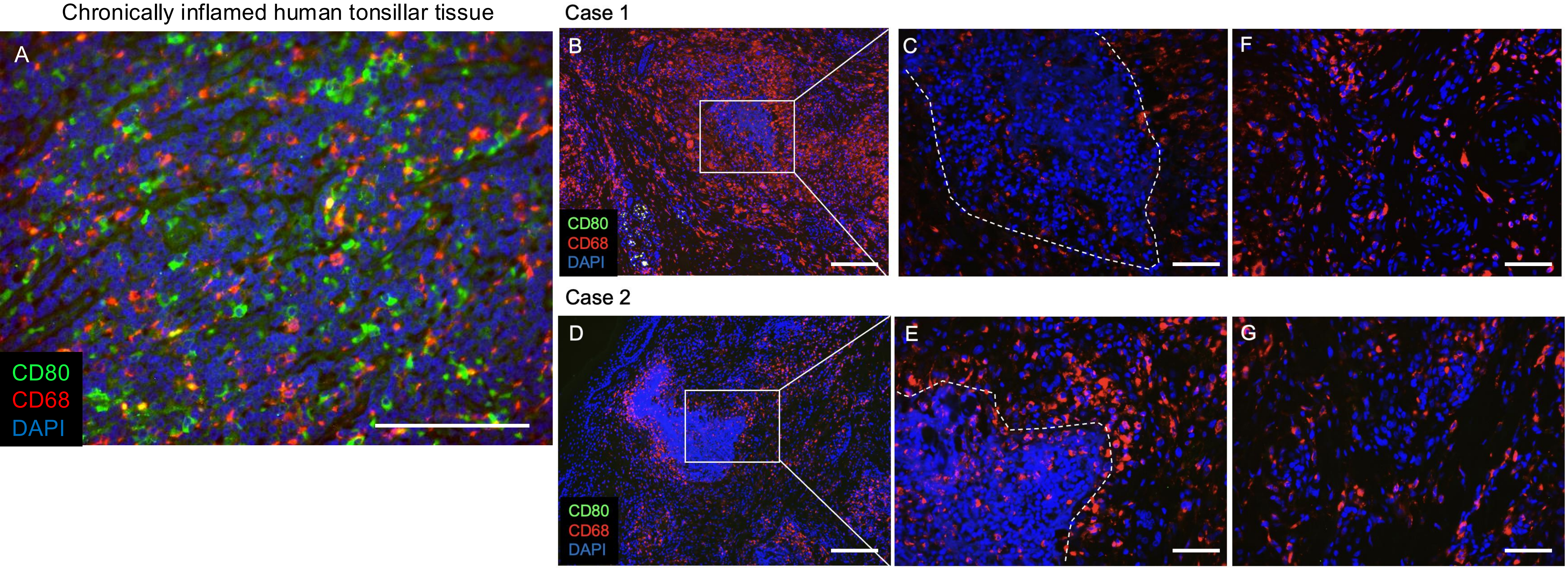

Supplementary Figure 4. Evaluation of CD80 expression in infiltrating macrophages. Representative immunofluorescence staining for CD80 (green) and CD68 (red), with DAPI nuclear counterstaining (blue). Chronically inflamed human tonsillar tissue was used as a positive control and showed CD80-positive cells, confirming that the anti-CD80 antibody worked appropriately (A). CD80 and CD68 staining was performed in lesional skin from case 1 (B and C) and case 2 (D and E), as well as in perilesional skin from case 1 (F) and case 2 (G). CD68-positive macrophages were present, whereas CD80 expression was undetectable in both lesional and perilesional skin. The boxed regions in B and D are shown at higher magnification in C and E, respectively. Dashed lines delineate the NET-rich neutrophilic aggregates. Original magnifications: (B and D) ×100; (A, C, E, F, and G) ×400. Scale bars, 100 μm. The following primary antibodies were used: a rabbit monoclonal anti-CD80 antibody (clone EPR1157(2); Abcam, Cambridge, UK) and a mouse monoclonal anti-CD68 antibody (clone 514H12; Bio-Rad Laboratories, Hercules, CA, USA). Samples were mounted and counterstained using VECTASHIELD Vibrance Antifade Mounting Medium with DAPI (catalog no. H-1800-10; Vector Laboratories, Newark, CA, USA).

Supplementary Figure 5

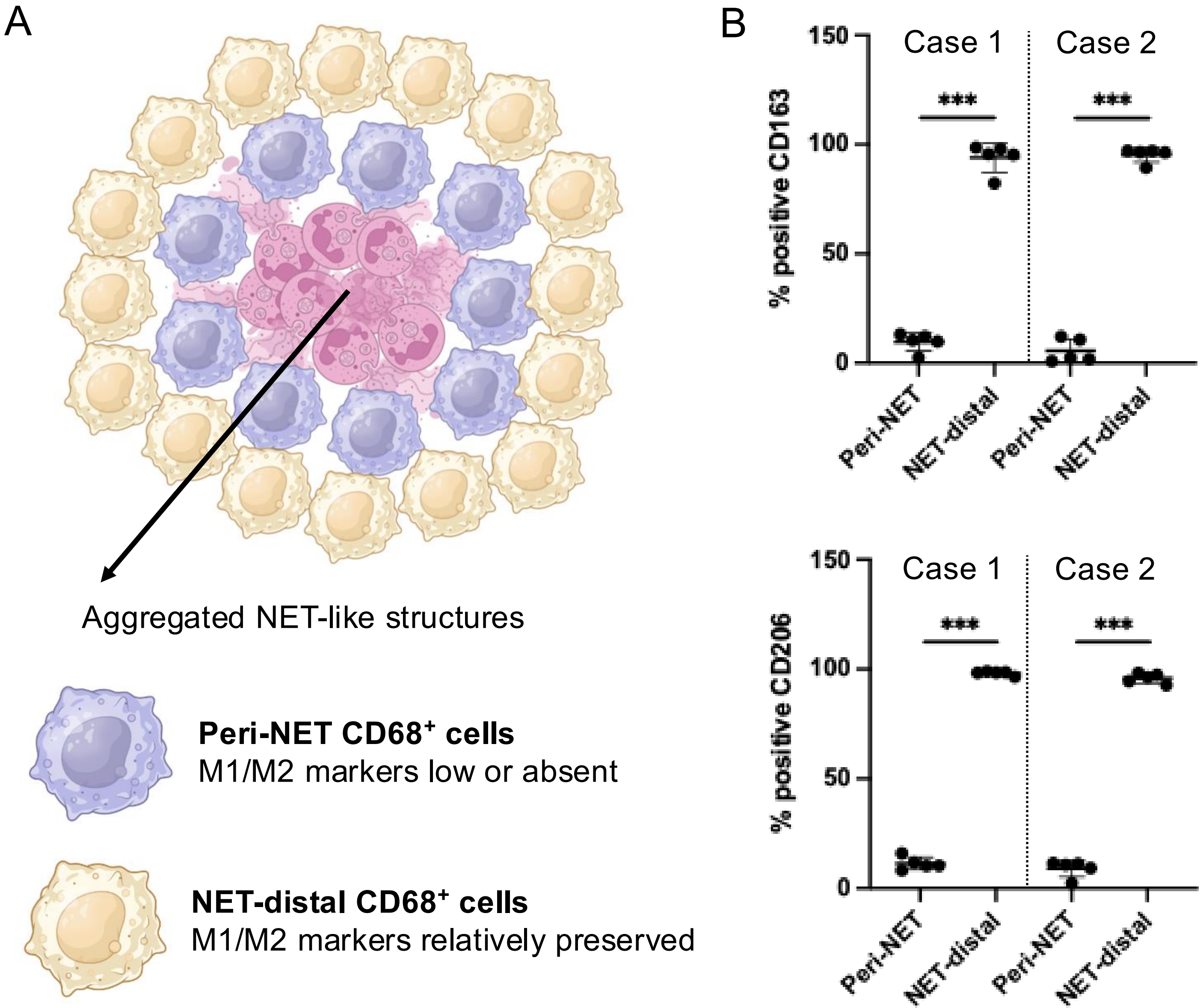

Supplementary Figure 5. Quantitative comparison of macrophage-marker expression between peri-NET and NET-distal cells. (A) Schematic definition of the regions used for quantification. Peri-NET CD68-positive cells were defined as cells immediately adjacent to dense NET-rich aggregates, whereas NET-distal CD68-positive cells were located farther from the aggregates. (B) Proportions of CD163-positive (upper) and CD206-positive (lower) cells among CD68-positive cells in the peri-NET and NET-distal regions of cases 1 and 2. Cells were counted in five ×400 fields per region in each case. Statistical comparisons were performed using Student's *t*-test. **$P < 0.001$. Panel A was created with BioRender.com.

Supplementary Figure 6

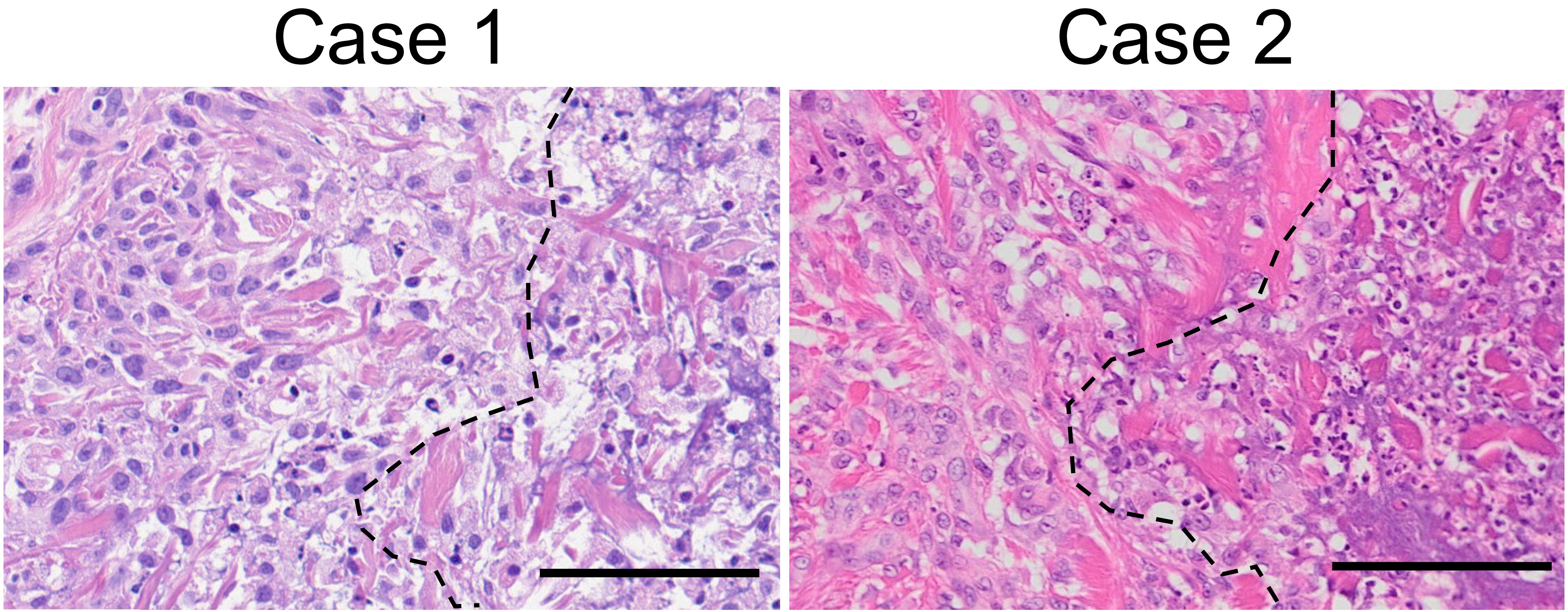

Supplementary Figure 6. Histological morphology of peri-NET cells in PNGD lesions. High-magnification hematoxylin and eosin–stained sections from Cases 1 and 2 show cells with histiocyte-like morphology surrounding the neutrophilic aggregates. The dashed lines delineate the boundary between the NET-rich neutrophilic aggregate (right) and the histiocyte-rich area (left). Scale bars, 100 μm.

Supplementary Figure 7

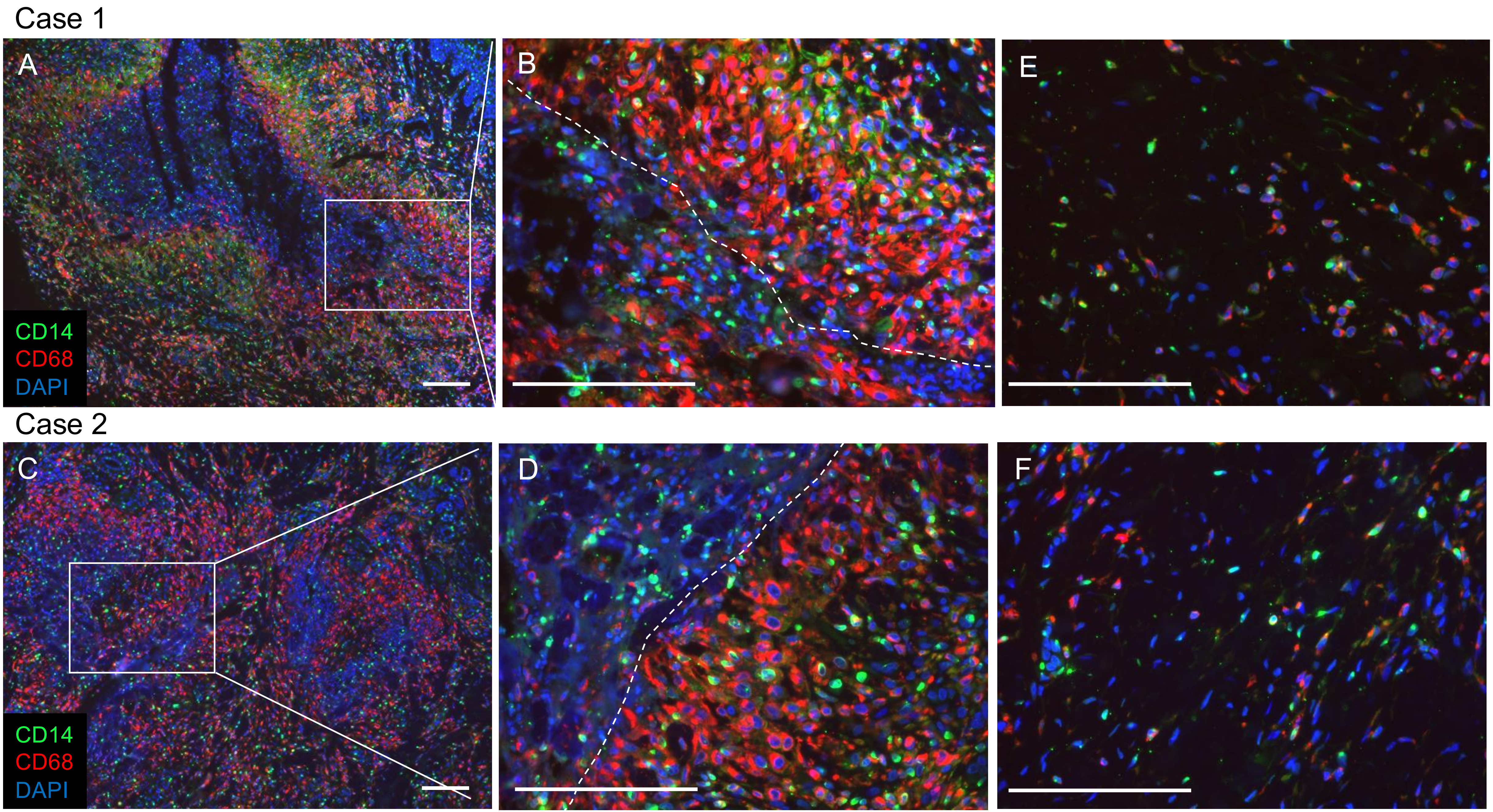

Supplementary Figure 7. CD14 expression in peri-NET cells. Representative immunofluorescence staining for CD14 (green) and CD68 (red), with DAPI nuclear counterstaining (blue), in lesional and perilesional skin from patients with PNGD. Low-magnification images of the lesional skin from case 1 (A) and case 2 (C) are shown, with the boxed regions presented at higher magnification in B and D, respectively. Dashed lines delineate the NET-rich neutrophilic aggregates. Perilesional skin from case 1 (E) and case 2 (F) is also shown. Original magnifications: (A and C) ×100; (B, D, E, and F) ×400. Scale bars, 100 μm. The following primary antibodies were used: a rabbit monoclonal anti-CD14 antibody (clone 001; Sino Biological, Beijing, China) and a mouse monoclonal anti-CD68 antibody (clone 514H12; Bio-Rad Laboratories, Hercules, CA, USA). Samples were mounted and counterstained using VECTASHIELD Vibrance Antifade Mounting Medium with DAPI (catalog no. H-1800-10; Vector Laboratories, Newark, CA, USA).

Supplementary Figure 8

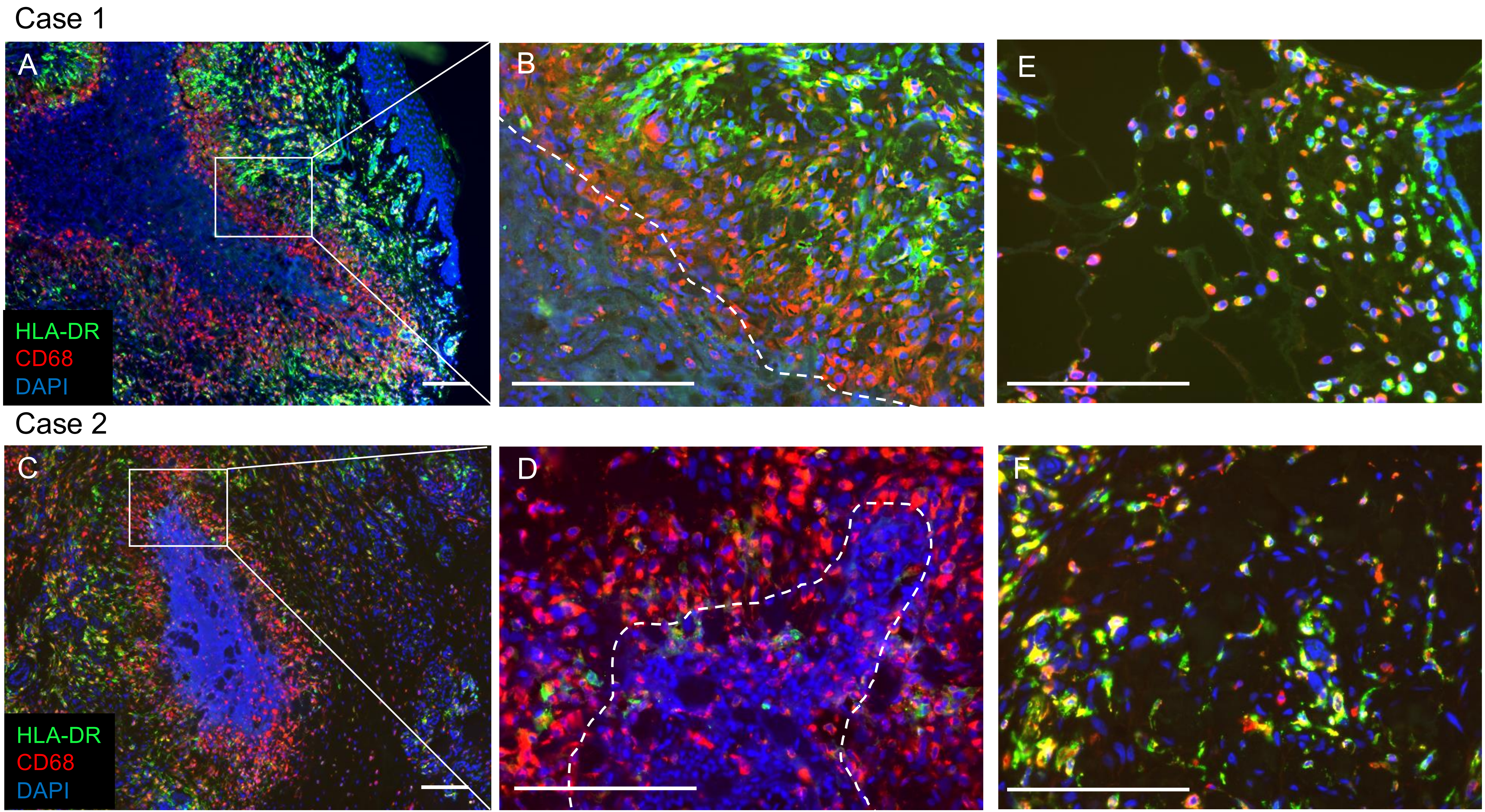

Supplementary Figure 8. HLA-DR expression in peri-NET cells. Representative immunofluorescence staining for HLA-DR (green) and CD68 (red), with DAPI nuclear counterstaining (blue), in lesional and perilesional skin from patients with PNGD. Low-magnification images of the lesional skin from case 1 (A) and case 2 (C) are shown, with the boxed regions presented at higher magnification in B and D, respectively. Dashed lines delineate the NET-rich neutrophilic aggregates. Perilesional skin from case 1 (E) and case 2 (F) is also shown. Original magnifications: (A and C) ×100; (B, D, E, and F) ×400. Scale bars, 100 μm. The following primary antibodies were used: a rabbit monoclonal anti-HLA-DR antibody (clone EPR3692; Abcam, Cambridge, UK) and a mouse monoclonal anti-CD68 antibody (clone 514H12; Bio-Rad Laboratories, Hercules, CA, USA). Samples were mounted and counterstained using VECTASHIELD Vibrance Antifade Mounting Medium with DAPI (catalog no. H-1800-10; Vector Laboratories, Newark, CA, USA).

Supplementary Figure 9

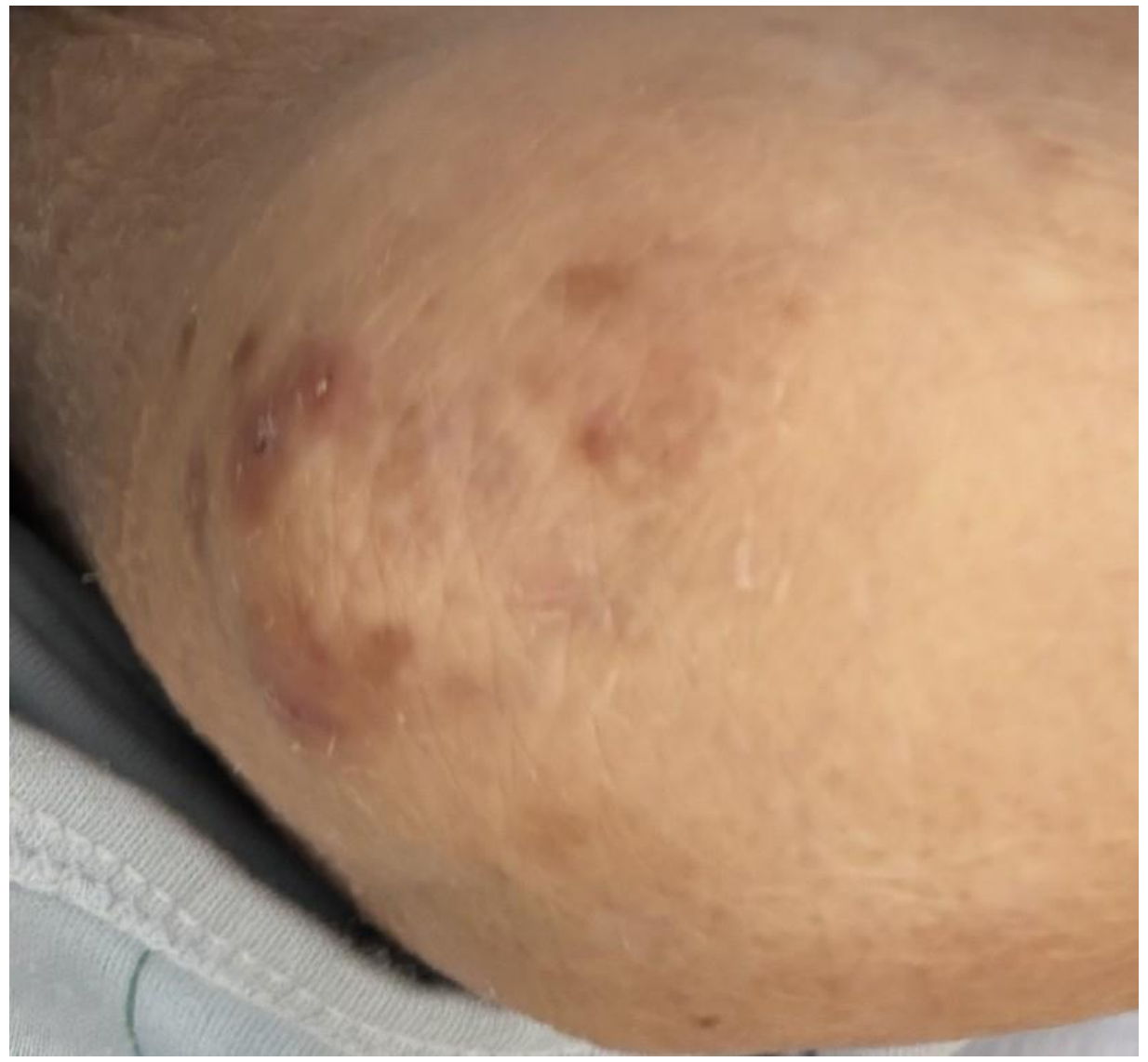

Supplementary Figure 9. Regression of the elbow lesions in Case 1 during bed rest. Clinical appearance of the elbow lesions after four weeks of hospitalization for a severe soft-tissue infection of the right lower extremity. During this period, the patient remained on bed rest and therefore avoided the habitual mechanical pressure on her elbows associated with wheelchair transfers. No treatment was administered specifically for the PNGD lesions. Compared with the initial presentation shown in Figure 1A, the erythematous papules and nodules on the elbow had markedly regressed.
